# Mycorrhizal symbiosis drives tolerance to potato cyst nematodes

**DOI:** 10.1016/j.isci.2026.114923

**Published:** 2026-02-06

**Authors:** M. Willow H. Maxwell, Aloka Hossain Fernando, Alex Papp, Chris A. Bell

**Affiliations:** 1 School of Biology, University of Leeds, Leeds LS2 9JT, UK; 2 Cornwall College, Eden Project, Bodelva, Cornwall PL24 2SG, UK

**Keywords:** Natural sciences, Plant biology, Interaction of plants with organisms, Molecular plant pathology, Agricultural science

## Abstract

Host plant tolerance to pathogens is increasingly relevant as resistance sources and control options become scarce. Arbuscular mycorrhizal (AM) fungi are known to enhance plant stress tolerance, but it remains unclear whether they are essential for, or complement, innate tolerance. We observed that potato cultivars described as tolerant to *G. pallida* suffered yield loss under nematode pressure when grown in sterile soils, indicating a lack of tolerance. The introduction of *Rhizophagus irregularis* increased tuber biomass during nematode parasitism, with cultivars commercially labelled as tolerant exhibiting a stronger response to AM fungi. The data suggest cultivar differences in mycorrhizal responsiveness with the differential expression of a range of plant sugar transporter genes in “tolerant” cultivars inferring a role of sugar allocation in host tolerance. Overall, AM fungi are critical for conferring tolerance against *G. pallida* and revealing the underpinning genes may provide useful targets to explore in current commercially desirable yet intolerant cultivars.

## Highlights

• Potato cultivars labeled as tolerant lose yield under nematode pressure in sterile soils
• Arbuscular mycorrhizal fungi improve tuber biomass during nematode parasitism
• Tolerant cultivars show stronger mycorrhizal responsiveness than intolerant ones
• Sugar transporter gene expression suggests a role in tolerance mechanisms

## Graphical abstract

**Figure:**
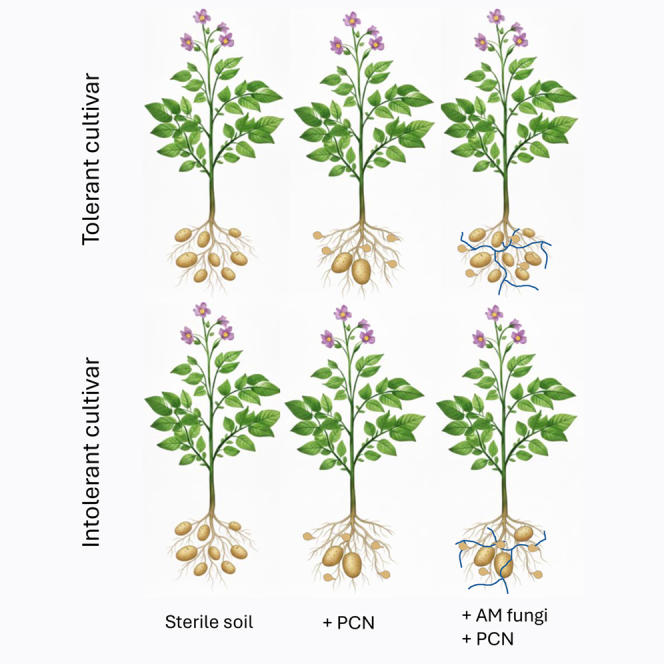

## Introduction

Globally, potato has been the most produced non-cereal food crop for over 50 years, with over 380 million tonnes produced in 2023.^1^ Like all crops, potato is subject to various pests and diseases, ranging from insects and nematodes to fungi and viruses. *Globodera pallida*, the white potato cyst nematode, is a widespread biotrophic endoparasite that, along with the closely related *G. rostochiensis,* causes major damage to potato crops worldwide (approximately 9% crop losses per annum^2^). Motile second-stage juveniles (J2s) hatch from their eggs in response to hatch-inducing compounds exuded by plant roots. Once hatched, J2s invade the root and establish specialized feeding structures, known as syncytia, that act as nutrient sinks from which the nematodes acquire resources, such as sugars.^3^ Upon feeding, the J2s moult through the juvenile stages and differentiate into male or female adults. As the gravid females senesce, their cuticles harden and tan, forming a toughened structure, termed a cyst. These egg-filled cysts may persist in the soil for decades until a new host crop is planted and the presence of fresh roots induces hatching of the second generation.^4^

In addition to plant-parasitic nematodes, plants can concurrently host mutualistic soil-dwelling symbionts, such as arbuscular mycorrhizal (AM) fungi. AM fungi can colonize the roots of approximately 70% of vascular plant species^5^ and over half of the plant species that are host to a plant-parasitic nematode are also hosts to AM fungi.^6^ Fungal hyphae can access inorganic nutrients, such as phosphorus and nitrogen, from the soil and exchange them with host roots for carbon-based compounds (e.g., sugars and fatty acids), which is often considered to lead to beneficial outcomes for both plant and fungi.^7^^,^^8^

The host-symbiont relation can thus range from parasitic (*G. pallida*) at one end of the spectrum to mutualistic (AM fungi) at the other. While AM fungi enhance potato nutrition and mitigate yield losses caused by *G. pallida,* the nematodes may also exploit this enhanced nutrition for their own reproduction.^9^^,^^10^ Tolerance (the plant’s ability to sustain growth and yield upon infection) and resistance (the plant’s ability to limit the reproduction and multiplication of the nematode) of potato cultivars to *G. pallida* are of great interest to growers’ pest management strategies.^11^ Tolerance and resistance are genetically and physiologically independent traits.^11^ For example, certain potato cultivars are known to be tolerant to *G. pallida*, i.e., their yield is unaffected by infection, while nematode reproduction rates may be even higher than on intolerant plants. The underpinning genetic mechanisms to this tolerance are not well understood due to a traditional focus of research upon resistance, novel approaches are being developed to expedite insights.^12^

Despite their different effects on host-plant health, plant-parasitic nematodes and mutualistic fungi both rely upon the host plant for the entirety of their organic carbon (C) intake, inevitably leading to competition for compounds, such as hexoses.^6^^,^^10^ AM fungi-colonized potato roots appear to store an increased abundance of sugars, yet also reduce their exchange with the fungus itself, upon concurrent *G. pallida* infection.^10^ This data suggests a systemic change in sugar translocation in response to parasitism, potentially to reduce losses, by diverting C away from parasitized roots and toward roots engaging with mutualists.

Sugars Will Eventually be Exported Transporter (SWEET) are highly conserved genes that encode transmembrane sugar efflux transporters in both plants and nematodes.^13^^,^^14^ Both pathogens and AM fungi are known to impact plant host SWEET gene expression, sometimes in a symbiont-specific manner.^10^^,^^13^ SWEET genes are suggested to participate in sugar transport to the nematode’s feeding site within plant vascular tissues.^15^ Combined, these findings implicate an important role for plant SWEETs in host resource allocation in response to symbiosis. It is unknown how SWEET genes may impact, or contribute toward, the tolerance and/or resistance of potato to *G. pallida.*

Here, we assessed the impact of AM fungal colonization on multiple, commercially available potato cultivars designated as varying in tolerance toward *G. pallida*. We explored the role of a range of SWEET genes, as part of a sugar transportation mechanism, in tolerance against this damaging pest. We show that AM fungi bolster tolerance regardless of the initial cultivar level or the extent of root colonization, and that the differential expression of a range of resource-transporting genes may enable the host to tolerate potential resource losses to the parasitizing nematodes.

## Results

### The impact of arbuscular mycorrhizal fungal-colonization on the tolerance of five potato cultivars toward Globodera pallida

**Figure 1:**
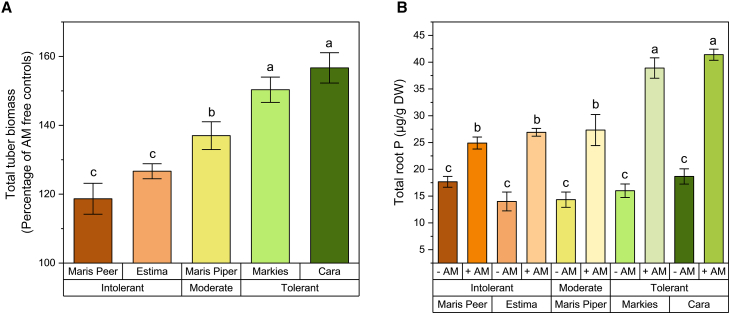
The impact of AM fungal-colonization on the tuber biomass and root phosphorus content of five potato cultivars (A) Tuber biomass was measured 16 weeks post-inoculation from cultivars that differ in their designated *G. pallida* tolerance (Maris Peer, Estima, Maris Piper, Markies, and Cara). Data from AM fungal colonized plants are presented relative to AM-free controls, i.e., *y* axis 100% is indicative of the tuber biomass of that specific cultivar in the absence of AM fungi. (B) The root P content was quantified and displayed per g dry weight root. Five biological replicates per treatment were assessed. Error bars represent standard deviation. Different letters denote significance where *p* < 0.05 (A, one-way ANOVA Tukey; B, two-way ANOVA Tukey).

After 16 weeks of growth, all five potato cultivars yielded greater tuber biomass when inoculated with AM fungi (Figure 1A; *p* < 0.05, two-way ANOVA Tukey, Factor one = AM fungal inoculation). The cultivar type also significantly impacted the tuber biomass upon harvest (*p* < 0.05, two-way ANOVA Tukey, Factor two = cultivar). Greater tuber biomass of AM-inoculated pots relative to uninoculated pots was obtained from cultivars commercially labelled as tolerant to *G. pallida*, compared to intolerant (factor one x factor two *p* < 0.05, two-way ANOVA Tukey).

Greater total P content was observed in the roots of AM-inoculated plants (two-way ANOVA, *p* < 0.05, Factor one = AM fungal inoculation), with no root P variance between cultivars without AM fungal inoculation (Figure 1B; two-way ANOVA, *p* > 0.05, Factor two = cultivar). A significant interaction between both factors suggests that AM fungi have a greater effect on the total P within the roots of commercially tolerant compared to intolerant cultivars (factor one x factor two *p* < 0.05, two-way ANOVA Tukey).

**Figure 2:**
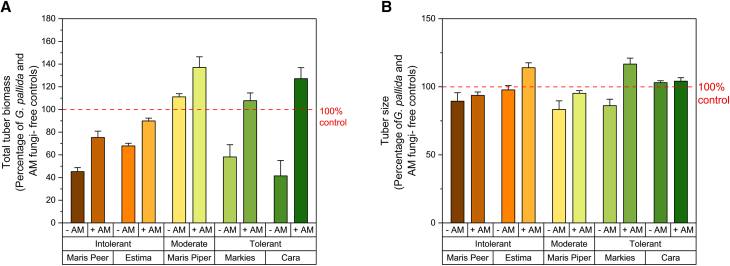
The impact of AM fungal-colonization on the tolerance of five potato cultivars towards *Globodera pallida* Tuber biomass was measured 16 weeks post-inoculation from cultivars that differ in their designated *G. pallida* tolerance (Maris Peer, Estima, Maris Piper, Markies, and Cara). The dashed red line represents the mean control tuber biomass (A) and tuber size (B) per cultivar when grown in sterile soils (controls), as 100%. Soils were inoculated with *G. pallida* (Gp) and AM fungi (AM), and their data are shown as a percentage of the control data. Five biological replicates per treatment were assessed. Error bars represent standard deviation.

The impact of concurrent *G. pallida* and AM fungal inoculation on tuber biomass of the five cultivars was measured to assess the role of AM fungi on potato tolerance. In the absence of AM fungi, *G. pallida* infection reduced tuber biomass by up to 60% compared to *G. pallida*-free, asymbiotic plants (Figure 2A; yields from each cultivar in sterile control soil are indicated by the red dashed line at 100%). *Globodera pallida* infection reduced the tuber biomass in both the intolerant (Maris Peer, 45% of control biomass; Estima, 68%), as well as the tolerant cultivars (Markies, 58%; Cara, 41%), compared to *G. pallida*-free control plants (*p* < 0.01, one-way ANOVA Tukey). Maris Piper was the only cultivar with a statistically similar tuber biomass in both control and *G. pallida*-infected plants, when grown in sterilized soil.

Colonization of *G. pallida-*infected roots by AM fungi resulted in an increased tuber biomass of all cultivars, compared to AM-free *G. pallida-*infected plants (Figure 2A; *p* < 0.01 two-way ANOVA Tukey, Factor one = AM fungal inoculation), regardless of their prescribed tolerance to *G. pallida*. The interaction between AM fungal inoculation and cultivar (*p* < 0.01 two-way ANOVA, AM inoculation x cultivar) suggests a differential response of AM fungal inoculation depending on cultivar identity when quantifying tuber biomass. AM fungal colonization of tolerant cultivars (Markies and Cara) resulted in a larger increase in total tuber biomass compared to intolerant cultivars (Maris Peer and Estima), when compared to AM-free *G. pallida*-infected plants. Specifically, AM fungal colonization increased tuber biomass of *G. pallida*-infected Markies from 58% to 108% of control plants, and of *G. pallida*-infected Cara from 41% to 127% of control plants, a 3-fold increase, outlining a stronger enhancement of tolerant vs. intolerant cultivars by AM fungal colonization.

AM fungal-colonization increased the tuber size of Estima and Markies (Figure 2B; *p* < 0.05 one-way ANOVA Tukey) but did not affect the other cultivars.

### The impact of arbuscular mycorrhizal fungal colonization on *G. pallida* across potato cultivars

**Figure 3:**
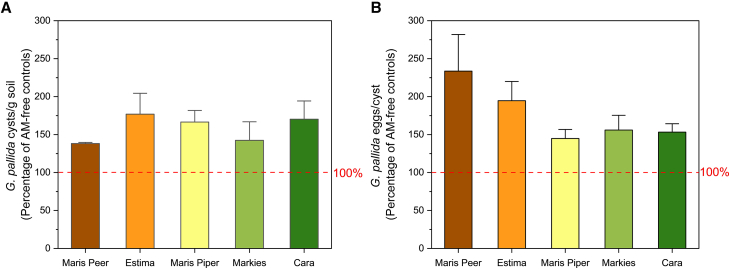
The effect of AM fungi on *Globodera pallida* reproduction across different potato cultivars with varying levels of tolerance Maris Peer, Estima, Maris Piper, Markies, and Cara were inoculated with *G. pallida* alone or concurrently with AM fungi. Post-harvest, (A) cysts were quantified, and (B) cyst egg content was determined. Data from AM-colonized plants is shown by the bars, which are calculated as a percentage of AM-free plants (shown by the red dashed line at 100%). Five biological replicates were sampled per treatment. Error bars represent standard deviation.

The *G. pallida* soil population at harvest was similar in soils collected from the different potato cultivars (Figure 3A). Across all five cultivars, AM fungal-colonization significantly increased the number of cysts post-harvest, compared to AM-free plants (Figure 3A; Two sample *t* test *p* < 0.01). We found no influence of cultivar type on the number of cysts recovered (Figure 3A; one-way ANOVA Tukey *p* > 0.05). Across all cultivars, we also observed a similar increase in eggs/cyst from plants co-colonized with AM fungi, with egg numbers increasing by 140–230% of those collected from AM-free plants (Figure 3B; Two sample *t* test *p* < 0.001). There was no effect of cultivar on the AM fungal-induced increase in *G. pallida* eggs/cyst (*p* > 0.05, one-way ANOVA Tukey) (Figure 3B).

### Arbuscular mycorrhizal fungal colonization rates of potato cultivars

**Figure 4:**
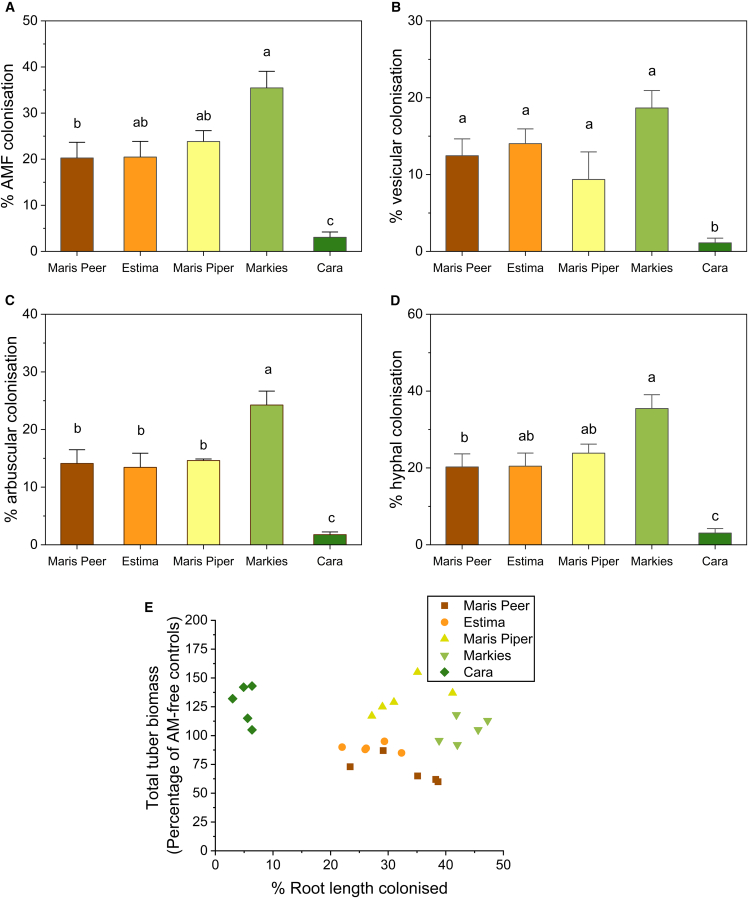
AM fungal-colonization rate of potato cultivars with varying levels of tolerance to *Globodera pallida,* and correlation to biomass Roots from *G. pallida*-infected Maris Peer, Estima, Maris Piper, Markies, and Cara were obtained six weeks post-inoculation with AM fungi. Root samples were stained in ink-vinegar solution, and AM fungal structures were counted at 200× magnification to quantify the percentage of colonization; (A) total root, (B) by arbuscules, (C) by vesicles, (D) by hyphae. (E) Correlation plot between AM fungal colonization and total tuber biomass from Figure 2A (Pearson’s r = −0.19 *p* = 0.37). Colonization rate is expressed as a percentage of the total root colonized. Five biological replicates were analyzed per cultivar. Different letters denote significance in A, B, C, and D, where *p* < 0.05 (one-way ANOVA, Tukey). Error bars represent standard deviation.

We observed AM fungal-colonization of roots of all tested potato cultivars, measured by percentage of root colonized; Maris Peer (20%), Estima (20%), Maris Piper (25%), Markies (35%), and Cara (5%) (Figure 4A). Total root colonization was greatest in the commercially designated tolerant cultivar Markies (*p* < 0.01 one-way ANOVA Tukey), while Cara (also tolerant) exhibited the lowest colonization (*p* < 0.01 one-way ANOVA Tukey). Furthermore, Markies contained the greatest abundance of arbuscules while Cara contained the lowest arbuscule, vesicle, and hyphal colonization rates (*p* < 0.01 one-way ANOVA Tukey) (Figures 4B, 4C, and 4D). There was no significant correlation between AM fungal colonization and tuber biomass increases for all cultivars (Figure 4E, Pearson’s r = −0.19 *p* = 0.37).

### Influence of arbuscular mycorrhizal fungal colonization and G. pallida infection on potto root sugars will eventually be exported transporter gene expression

Due to the prominent role of plant SWEET genes in host-AM fungal interactions and of host sugar translocation in tolerance to biotic stresses,^16^ we investigated the effect of AM fungi and *G. pallida* on the role of plant SWEET sugar transporters across the five potato cultivars. No amplification could be obtained for any tested SWEET gene from the roots of Maris Peer and Cara using published primer sets,^17^ leading to the design of new primers for this study.

**Figure 5:**
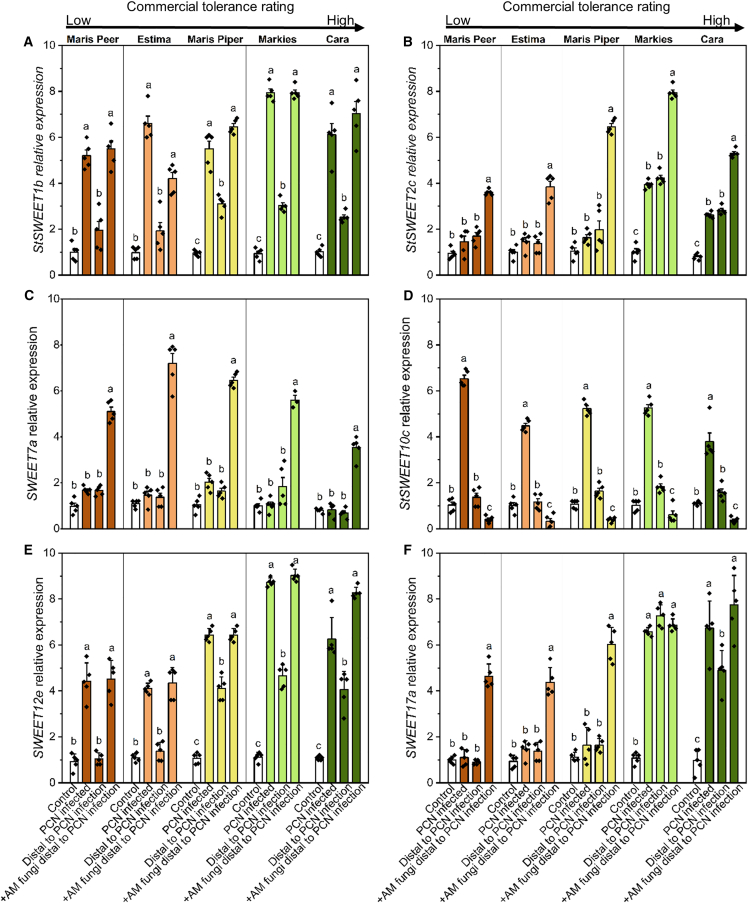
Root SWEET gene expression profiles in response to *Globodera pallida* and AM fungi Potato cultivars with varying levels of tolerance to *G. pallida* were chosen: Maris Peer, Estima, Maris Piper, Markies, and Cara. Root sections were taken from uninfected control plants, *G. pallida* (PCN) infected tissue, asymbiotic root distal to *G. pallida* infection, and AM-colonized root distal to *G. pallida* infection. Relative expression was quantified via the ^ΔΔ^Ct method for six SWEET genes; *SWEET1b*, *2c*, *7a*, *10c*, *12e*, and *17a*. Samples were assessed in technical triplicate and five biological replicates, relative to Control samples. Letters (a, b, c) denote significance between root section types within each cultivar for each gene (one-way ANOVA, Tukey *p* < 0.05). Error bars represent the standard error of the mean. The commercial tolerance description of each potato cultivar is illustrated at the top of the figure.

The Clade I gene *SWEET1b* was upregulated locally to *G. pallida*-infected root tissue in all five cultivars. The induction of this gene appeared to be systemic when AM fungi were colonizing the same host, if AM fungi were absent, then the greatest induction was limited to the nematode-infected tissue (Figure 5A). *SWEET2c*, a second CLADE I gene, was induced locally and distally to *G.* *pallida infection* in Markies and Cara (commercially designated as tolerant), with a stronger induction if tissues distal to nematode infection were colonized by AM fungi (Figure 5B). Furthermore, *SWEET2c* expression was increased in AM-colonized root tissue distal to *G. pallida* infection from all cultivars. In all five cultivars, *SWEET7a* (Clade II) was specifically upregulated in AM-colonized root tissue distal to *G. pallida* infection (Figure 5C), while *SWEET10c* (Clade III) was only upregulated in tissue local to *G. pallida* infection and was downregulated in distal AM-colonized roots (Figure 5D). *SWEET12e*, Clade III, was induced by local *G. pallida* infection in all five cultivars, with upregulation by approx. 8-fold in the tolerant cultivars (Figure 5E). In roots distal to *G. pallida* infection, *SWEET12e* was not upregulated in intolerant cultivars unless there was AM fungal colonization. While Maris Piper, Markies and Cara showed the expression of *SWEET12e* in distal root tissues absent of AM fungi, there was a further increase in expression if the fungus was present (Figure 5E). A Clade IV gene, *SWEET17a*, was upregulated in all five cultivars upon AM-colonization during *G. pallida* infection and systemically induced in the roots of the tolerant cultivars, Markies and Cara (Figure 5F).

## Discussion

The ability of a host to yield in the presence of a pest or pathogen, i.e., tolerance, is a relatively ill-defined area of plant-pathogen interactions. The results from our glasshouse experiments suggest that soil microorganisms, specifically AM fungi, contribute toward the tolerance of potato plants toward *Globodera pallida*. We observed that colonization by AM fungi, regardless of the colonization rate, resulted in a systemic increase in sugar transporter gene expression in potato roots. High expression of these genes in tolerant cultivars may enable elevated resource transport or nutrient uptake from the soil in response to *G. pallida* infection. This study proposes that AM fungi may mediate tolerance by inducing transcriptional changes that enable resources to offset those lost to feeding nematodes and that host tolerance may indicate, at least to some degree, host responsiveness to these mutualists.

*Globodera pallida* infection resulted in reductions in tuber biomass of over 50% when grown in sterile soils, regardless of the cultivar’s commercial tolerance designation, i.e., we observed no indication of a “tolerant” cultivar in sterile soils. AM fungal inoculation of sterile soils increased tuber biomass to provide a degree of tolerance toward *G. pallida*, consistent with observed effects of AM fungi on other nematode and host species.^18^ Interestingly, when plants were infected with *G. pallida*, co-colonization with AM fungi had a relatively small positive effect on tuber biomass for the commercially designated intolerant (average tuber biomass increase by 1.5-fold) and moderately tolerant cultivars (average tuber biomass increase by 1.2-fold), while dramatically increasing tuber biomass of the commercially designated tolerant cultivar, Cara (average tuber biomass increase by 3-fold). As the tolerance designations of these potato cultivars have come from field reports, as opposed to sterile soil-based experiments, they describe tolerance in the field environment, which would likely contain a plethora of organisms, including AM fungi. The observed lack of tolerance in sterile soil, particularly for the cultivars that are designated as tolerant, suggests that tolerance ratings are perhaps inclusive of, and describe cultivar responsiveness to, the soil microbiome. For these cultivars, we suggest that tolerance is, in fact, AM-facilitated tolerance. Maris Piper, which is sold as a moderately tolerant cultivar, produced similar tuber biomass under AM-free conditions when inoculated with *G. pallida* compared to nematode-free plants, suggesting intrinsic tolerance. This indicates that some cultivars may possess baseline tolerance that AM fungi can enhance, rather than AM fungi being essential for its induction. Further studies are required to elucidate plant tolerance to pathogens before we can dissect innate and AM-induced tolerance mechanisms.

Within this study, we tested the model AM fungus, *Rhizophagus irregularis,* other fungal species may give different responses, such as enhanced benefits to host tolerance or perhaps even detrimental effects on the host. The responsiveness of wheat to AM fungi was found to be linked to varietal soil P-uptake efficiency.^19^ Here, our data showed that potato cultivars with enhanced tolerance to *G. pallida* also contained more root P when colonized by AM fungi, suggesting a potentially greater responsiveness of these cultivars to AM fungi. The greatest P content was observed within the commercially labelled tolerant varieties, Markies and Cara; however, Markies exhibited the greatest percentage root colonization and arbuscule number by the fungus, while Cara had the lowest. Overall, this suggests that tuber biomass increases and root P content are not directly related to the amount of root colonized by the fungus.

These findings suggest that the protection from losses due to *G. pallida* infection that AM fungi offer is not linked to AM fungal colonization rate, consistent with other reports of AM function-colonization rate independence.^20^^,^^21^ Greatest AM fungal colonization of Markies roots would support a prominent role of colonization levels in potato tolerance; however, contrastingly, the low colonization rate of Cara, coupled with its apparently large responsiveness to this low colonization level, suggests that the degree of tolerance and colonization levels are not linked. This suggests a potential minimum threshold of colonization that provides AM-induced benefits, and that some cultivars can exhibit a greater response to a relatively reduced fungal association. This AM-responsiveness may be what makes a cultivar *G. pallida*-tolerant in the field. Although not tested here, morphological differences in root diameter, length, and depth may factor into cultivar tolerance, as plants with deeper roots can access more mobile soil nutrients^22^ and thicker roots have more space for AM fungal structures. Root diameter was previously found to be the best predictor for AM fungal colonization rate in diverse plant species.^23^^,^^24^ Root morphology was not mapped in this study, but Cara (tolerant) has a larger root system than Maris Piper (moderate) and Estima (intolerant), with Estima having the smallest of these three.^25^

While AM fungi enhanced host-plant tolerance to *G. pallida*, they also increased *G. pallida* populations, similar to previous findings.^9^^,^^10^ Our data are consistent with the cultivars’ uniform resistance score of 2 (scale is 1–9 susceptible-resistant^26^^,^^27^). Therefore, AM fungi may only provide short-term benefits for potato production. We saw an increase in the number of cysts/g soil and also the number of eggs/cyst in AM-colonized plants regardless of their tolerance designation. This suggests that while resource flow and uptake may contribute to tolerance, which is limited by host resources, it does not directly link to resistance, which has been observed in other systems.^28^ In fact, the altered nutrition of potato by AM fungi has been previously shown to increase the nutrition of nematodes parasitizing the same root system, allowing greater parasite reproduction.^9^ As the addition of AM fungi to a field soil to boost potato tolerance to *G. pallida* may lead to a more rapid increase in *G. pallida* numbers, it becomes crucial to develop potato cultivars that are tolerant as well as resistant to *G. pallida* for AM fungi-induced tolerance to have any viable agricultural applications. This highlights the importance of better understanding and mapping the genetic mechanisms behind AM-induced tolerance in the tested cultivars and combining these findings with knowledge regarding *G. pallida-*resistance in potato.^29^ Epidemiological modeling shows how tolerance traits in general, while beneficial to individual yield, have negative implications through allowing pathogen proliferation that can promote their spread across cropping systems. By contrast, resistance reduces disease across the crop community, generating positive spillover benefits to other growers. This supports the need to develop potato cultivars that combine AM fungi-induced tolerance with genetic resistance, to maintain plant vigor while curbing *G. pallida* population growth - avoiding the potential spiral of tolerance-driven nematode increases and negative implications on field ecology as well as society.^30^

AM fungi induce a multitude of transcriptional as well as physiological changes in their host roots. In potato, AM fungi induce the differential expression of SWEET genes throughout the root system.^17^ Additionally, plant tolerance to pests and pathogens is known to be linked to resource availability.^28^^,^^31^ Pathogen-induced SWEET upregulation has previously been described in *Arabidopsis thaliana* and in crop plants such as rice, cassava, orange, and grape.^13^ In all of these cases, SWEET upregulation leads to sugar transport to the pathogen in question. However, in sweet potato, fungal infection can lead to the overexpression of the clade III SWEET gene *IbSWEET10,* and this restricts the flow of sugar to the fungal pathogen, thus limiting the plant’s loss of resources to this infection.^32^ SWEET genes can play various roles in plant-pathogen or plant-symbiont interactions, and many of these interactions are not yet well understood.

Here, we quantified the expression of six SWEET genes in root tissue from different potato cultivars that were either directly infected by *G. pallida* or tissue that was distal to *G. pallida* infection with or without AM fungi. Genes were selected due to their known expression in AM-colonized root tissue.^17^ Overall, we observed that *SWEET1b, 2c, 7a, 12e* and *17a* were upregulated in AM-colonized root sections. The commercially designated tolerant cultivars, Markies and Cara, often exhibited high expression of these genes, particularly in AM-colonized tissue. Consistent with previous reports, *SWEET7a* was specifically expressed in AM-colonized tissues^17^ and appeared unaffected by *G. pallida*.^10^ Strong upregulation of *SWEET1b* in AM-colonized plants is consistent with previous observations in *Medicago truncatula,* where *SWEET1b* is localized to arbuscule-containing root cells, inferring a role in the transportation of glucose across the peri-arbuscular membrane for uptake by the fungus.^33^ This role may be pertinent in our system, where we hypothesize that systemic sugar transport, or import, and usage is pivotal in host tolerance to nematode parasitism and response to infection, as seen in *Fusarium oxysporum* infection in sweet potato^32^ and *Pseudomonas syringae* in *Arabidopsis*.^34^ Additionally, a second hexose-transporting SWEET, *2c*, was upregulated in Markies. In other systems, *SWEET2c* is proposed to reduce loss of sugars to the rhizosphere, thereby linked to host performance and, potentially indirectly, to tolerance (Chen et al. 2015). However, evidencing the fine balance within the plant, a loss-of-function mutation in *SWEET2* increases the susceptibility of *Arabidopsis thaliana* to *Pythium* infection.^35^ This suggests that although tolerance and resistance may not be directly linked, attempting to modulate one may have indirect effects on the other.

*SWEET10c* was specifically induced in *G. pallida-*infected tissue, inferring it as a potential candidate as a gene targeted by the pathogen and not the mutualist. This could possibly be a future susceptibility gene to be targeted to induce resistance, similar to broad-spectrum SWEET-based resistance engineering in rice against *Xanthomonas oryzae*.^36^ Interestingly, the *G. pallida*-responsive *SWEET10c* was downregulated by AM fungi, consistent with previous research that suggested AM fungal-downregulated SWEET genes form a *Solanum-specific* subclade within Clade III.^17^

The observed large upregulation of *SWEET17a* in *G. pallida* feeding sites and AM-colonized roots of tolerant potato cultivars proposes this gene as an interesting candidate for future study due to its role in exporting fructose from the vacuole for plant metabolism in other systems.^37^ We hypothesize that the use of vacuolar material may be more dynamic in tolerant cultivars, maintaining the plants’ sugar levels upon loss due to infection. Alternatively, *SWEET17a* regulation may be an attempt to limit vacuolar leakage into spaces where the sugars may feed the pathogen,^38^ albeit in vain for the nematode pest used in our study. Functional studies of these genes across cultivars, and ideally across multiple pathogens, are now required to strengthen our understanding of their role in pathogen tolerance.

Overall, AM fungi appear to be necessary for host tolerance to *G. pallida*, facilitating the expression of a tolerant phenotype from a tolerant genotype, under the tested growing conditions. We observed a lack of tolerance to *G. pallida* in sterile soils in potato cultivars designated as tolerant by growers. Promoting the diversity of soil organisms may therefore have direct implications on host tolerance to *G. pallida*. We found a general increase in host SWEET gene expression in AM-colonized tissues, with strong upregulation observed in commercially designated tolerant cultivars, inferring the potential role of sugar usage and transportation in host tolerance. Determining which genes may be associated with the beneficial outcomes of host-AM interactions may provide targets to explore in commercially desirable yet intolerant potato cultivars. The tolerance that AM fungi can induce in these potato cultivars is promising for field applications, but the enhanced nutritional status AM fungi confer to the host plant also comes with the downside of increased *G. pallida* fitness. Over time, this may lead to *G. pallida* populations that even tolerant plants cannot sustain. More research into coupling *G. pallida* tolerance with resistance in potato plants is necessary.

### Limitations of the study

Our experiments were conducted under controlled conditions using sterilized soils and a single AM fungal species, which may not fully represent the complexity of field environments where diverse microbial communities and variable soil properties influence plant-nematode interactions. Further research with different or mixed fungal species inocula is needed to determine wider effects. Our study focused on a limited number of potato cultivars and one nematode species, so the generality of these findings across broader germplasm and other cyst nematodes remains to be determined.

## Resource availability

### Lead contact

Further information and requests for resources should be directed to the lead contact, Chris A. Bell c.a.bell@leeds.ac.uk.

### Materials availability

This article does not report original code.

This study did not generate unique reagents.

### Data and code availability

All data reported in this article will be shared by the lead contact upon request. Code: This article does not report original code.

## Acknowledgments

C.A.B. is supported by a 10.13039/501100000268BBSRC Discovery Fellowship (BB/X009823/1) and a Michael Beverley Innovation Fellowship. We thank Prof. P. Urwin for hosting these fellowships in his laboratory space. We thank The School of Biology, University of Leeds, for supporting M.W.H.M. A Generation Research studentship supported A.H.F, and a BSPP studentship supported A.P. We thank Vegetable Consultancy Services and Dr. J. Fortune for providing potato cultivars.

## Author contributions

M.W.H.M., A.H-F., and A.P. conducted the research. C.A.B. and M.W.H.M. designed the research, analyzed the data, and wrote the article.

## Declaration of interests

The authors declare no competing interests.

## STAR★Methods

### Key resources table

**Table:** 

REAGENT or RESOURCE	SOURCE	IDENTIFIER
**Chemicalsandkits**		

Acetic acid - glacial	VWR	8187552500
Lactic acid 90%	Acros	189870010
Potassium Hydroxide	Acros	134060010
Pelikan Brilliant Black ink	NA	NA
Poly(vinyl alcohol)	Sigma	363146
Glycerol	Acros	158920025
E.Z.N.A.® Plant RNA Kit	Omega BioTek	R6827-02
iScript™ cDNA Synthesis Kit	Bio-Rad	1708891
Brilliant III Ultra-Fast SYBR® Green Master Mix	Agilent Technologies	600883

**Experimentalmodels:organisms/strains**		

*Solanum tuberosum* cv. Maris Peer, Estima, Maris Piper, Markies, Cara	Vegetable Consultancy Services	NA
Mycorrhizal inoculum	PlantWorks, UK	NA

**Software andalgorithms**		

R Studio 2025.10.01		RStudio, Inc

### Experimental model and study participant details

Potato (*Solanum tuberosum*) of five commercially available cultivars with different designated levels of tolerance to *G. pallida* (Table 1) were grown from tubers for 16 weeks, one chitted/sprouted tuber per pot, in ten-litre pots containing sterilised 50:50 sand:topsoil (RHS Silver Sand:Bailey Norfolk Topsoil). All soil was heat sterilised, a method previously shown not to affect plant growth.^9^^,^^10^^,^^39^ Heat sterilisation was achieved by heating the soil mix to 82 °C for two hours before allowing to cool prior to planting, according to the manufacturer’s instructions (ThermoForce Ltd, UK). Soil nutrients have been previously quantified after sterilisation by this method and provided ample nutrition for potato growth.^40^ The five potato cultivars were selected due to their similar resistance levels to *G. pallida* (Table 1). The *G. pallida* stock was bulked up previously on *S. tuberosum* cv. Désirée plants, in 50:50 sterilised sand:topsoil with *G. pallida* cysts (population Lindley) and quantified using Fenwick’s (1940) method^41^ before confirming egg viability via a hatching assay. This stock soil was thoroughly mixed into the sterilised sand-topsoil to obtain a final density of 10 eggs/g soil. This soil was then used as a *G. pallida* treatment. For AM fungal treatments, AM fungal inoculum was applied to the base of each tuber upon planting (approx. 44 g of *Rhizophagus irregularis* inoculum consisting of approx. 7, 000 spores plus hyphae, PlantWorks Limited, UK). All AM-free plants received a blank inoculum, consisting of pumice and zeolite (1:1) (the carrier substrate) minus the fungal content, to control for any available nutrients in the substrate. Blank carrier had been sterilised by the supplier. All treatments had five biological replicates for each cultivar.

**Table 1:** Tested potato cultivars and their tolerance and resistance to *Globodera pallida*

Cultivar	Tolerance	Resistance (score)
Maris Peer	Very intolerant^a^	Susceptible (2)^a^
Estima	Intolerant^a^	Susceptible (2)^a^
Maris Piper	Moderately tolerant^a^^,^^b^	Susceptible (2)^a^
Markies	Tolerant^b^	Susceptible (2)^a^
Cara	Very tolerant^a^	Susceptible (2)^a^

a Data sourced from Blok, Jones & Sharma (2023).

b Data sourced from Keer (2007). *Globodera pallida* resistance is scored on a 1-9 (susceptible-resistant) scale.

Pots were placed in a containment glasshouse under controlled conditions: constant temperature of 19 ± 1 °C and a 16-h photoperiod in a random layout and watered with 400 ml of water every other day. Plants were grown until senescence, approximately 16 weeks, to enable *G. pallida* reproduction and tuber development. Soil was kept for *G. pallida* quantification. Tubers from each plant were measured and weighed.

Potato plants of the five cultivars were also grown from tubers for six weeks in three-litre pots, with five biological replicates per cultivar per treatment. Treatments consisted of i) controls without either *G. pallida* or AM fungi, ii) *G. pallida* application at two weeks post-planting, iii) *G. pallida* application at two weeks post-planting in AM fungal-inoculated soil. Inoculation with AM fungi was as described above and upon initial tuber planting. For treatment ii) and iii), two-week-old plants were inoculated with 2000 *G. pallida* second-stage juveniles per plant. Hatched juveniles were used for infection rather than cyst-containing soil, as previously described, to reliably obtain samples from infected root tissue during the specific timeframe in which nematode feeding occurs, allowing more accurate interpretation of gene expression data. Four weeks post-infection, the roots were very carefully cleaned with tap water and a subsample taken for quantification of AM fungal colonisation. Remaining roots were laid out under a microscope and 50 one cm sections of root with visible nematode infection were collected and stored in aliquots at -70°C. Additionally, 50 sections of root without nematodes were collected and separately stored at -70°C. Similar sections were recovered from AM-colonised roots to overall yield sections from i) asymbiotic roots, ii) *G. pallida* infected tissue, iii) tissue distal to *G. pallida* infection, iv) AM-colonised tissue distal to *G. pallida* infection. These samples were used for RNA extractions, described below. It was not possible to reliably obtain sections from one cm sections containing both AM fungi and *G. pallida* due destructive staining methods required to confirm fungal colonisation.

### Method details

#### Quantification of root phosphorus content

Total phosphorus (P) content of plant material was determined using an adapted method.^42^ Forty-five mins post-acid digest of root samples,^9^ sample optical density was recorded at 822 nm using a spectrophotometer. A standard P solution was used to produce a standard curve against which total sample P was calculated.

#### Quantification of *G. pallida* reproduction

Upon harvesting of the ten-litre pots, the roots were shaken vigorously in the pots to ensure all mature cysts remained in the soil. The soil was then thoroughly mixed and the number of cysts/g soil was determined using Fenwick’s (1940)^41^ extraction method on three 100 g samples of soil from each pot. To quantify the reproductive capacity of the nematodes under each treatment, the number of unhatched second-stage juveniles per cyst was counted for ten extracted cysts per pot.

#### Quantification of fungal colonisation of roots

Every plant inoculated with AM fungi was confirmed to be colonised before analysis as well as the confirmation of no AM fungal colonisation of non-inoculated plants. This also validated heat sterilisation methods. A portion of root from each plant was stained using the ‘ink and vinegar’ method,^43^ as previously optimised for potato.^9^^,^^44^ Specifically, root samples were washed in water, cleared with 10 % KOH (w/v) for 30 min in a 90 °C heat block, and AM fungi structures were subsequently stained with the ink and vinegar mix (5 % Pelikan Brilliant Black, 5 % acetic acid, 90 % dH_2_O)^43^ for 15 min in a 90 °C heat block. Root samples were de-stained in 1% acetic acid, cut into sections and mounted on microscope slides using polyvinyl lacto-glycerol (16.6 g polyvinyl alcohol powder, 10 ml glycerol, 100 ml lactic acid, 100 ml _dd_H_2_O). Arbuscular, vesicular, hyphal and total root colonisation were quantified using the magnified intersection methodology (minimum of 100 intersects per plant, 200X magnification).^45^

#### RNA extraction and RT-qPCR

RNA was extracted from root tissue from asymbiotic control plants, *G. pallida* infected roots, distal roots to *G. pallida* infection, and AM-colonised roots distal to *G. pallida* infection (E.Z.N.A.® Plant RNA Kit; Omega BioTek, 2018) following the manufacturers protocol. RNA concentration was determined using a Nanodrop Lite Plus spectrophotometer (Thermo Fisher Scientific) and 500 ng was used for cDNA synthesis using iScript™ cDNA Synthesis Kit (Bio-Rad), according to the manufacturer’s instructions. The resulting cDNA was diluted 1:20 with nuclease-free water and stored at −20 °C.

Relative expression levels of six SWEET genes (Table 2) were quantified in the root sections through RT-qPCR using Brilliant III Ultra-Fast SYBR® Green Master Mix (Agilent Technologies, CA, USA). These genes were selected due to reported responsiveness to AM fungi in roots,^10^^,^^17^ as well as representing multiple SWEET clades (Table 2). Thermal cycling conditions consisted of 95 °C for 3 min, followed by 39 cycles of 95 °C for 15 s, 60 °C for 30 s, and 72 °C for 30 s. Elongation factor 1-alpha served as reference gene for normalisation. All primers were newly designed in this study due to a lack of amplification by previously published primer sets with these potato cultivars.^17^ Three sets of primers for each gene were designed through Primer-BLAST (NCBI) to ensure target gene specificity. All primer sets were assessed for amplification efficiency and a single primer set for each gene, which had an efficiency of 95-100 %, was used to quantify gene expression levels within sample tissues (Table 3). Technical triplicates were averaged to provide a biological replicate. Five biological replicates were conducted for each treatment. The relative gene expression was calculated using the 2 ^(ΔΔCt)^ method, with fold changes determined relative to control asymbiotic roots.^46^

**Table 2:** SWEET genes for expression analysis during *Globodera pallida* infection and AM fungal colonisation of potato roots

Gene	Phylogenetic clade	Inferred substrate
*StSWEET1b*	Clade I	Hexoses, specifically glucose
*StSWEET2c*	Clade I	Hexoses, specifically glucose
*StSWEET7a*	Clade II	Hexoses
*StSWEET10c*	Clade III	Sucrose
*StSWEET12e*	Clade III	Sucrose
*StSWEET17a*	Clade IV	Fructose

Phylogenetic data from Manck-Götzenberger & Requena, 2016.

**Table 3:** Primers designed and used in this study (5’-3’)

Gene	Forward primer	Reverse primer
*StSWEET1b*	GGAGCAGGACTTGAGGCATT	TGCCATGGAGAGCTAACATTGA
*StSWEET2c*	TTGATCTGGAACTCCGACGC	AGCTGGAGAGGGACAGGAAA
*StSWEET7a*	GCTGGGGCTAGCACAACTAA	ACTAGCGCAGCCTCGTTG
*StSWEET10c*	TGAGAATGTGCCCAAGTTGC	GGGGCTTGCTTTGTCAGTCA
*StSWEET12e*	ATCCAAGGAGGCTAGGGTAAGT	GCCACTGCAATCAACGTGTA
*StSWEET17a*	TGGTTTCCTTACCGCAGGTT	CGGGATACATGCGGGATACA
EF 1-alpha	ATTGGAAACGGATATGCTCCA	TCCTTACCTGAACGCCTGTCA

### Quantification and statistical analysis

Statistical analysis was conducted, and plots were generated, using OriginPro (OriginLab Corporation. Version 2024). One-way ANOVA and Tukey post hoc tests were used for comparisons within each cultivar across treatments. Two-way ANOVA was applied to assess the main effects of AM inoculation and cultivar, and their interaction, on response variables. In Figures 1, 2, and 3 the control (sterile soil) treatment values were used to normalise and visualise the treatment data on a percentage scale, e.g. tuber biomass per cultivar obtained in sterile soils are represented as 100 %, with AM fungi and *G. pallida* treatments then normalised to this 100 % value. In Figure 4, Pearson Correlation was used to determine the relationship between AM fungal colonisation and tuber biomass.
